# Household exposure, demographic and health characteristics associated with SARS-CoV-2 infection in a cohort study in Northern France

**DOI:** 10.1038/s41598-025-26851-y

**Published:** 2025-11-28

**Authors:** Simon Galmiche, Laurie Pinaud, Laura Tondeur, Stephane Pelleau, Mikaël Attia, Sandrine Castelain, Isabelle Staropoli, Cécile Duru, Aymar Davy Koffi, Sandrine Fernandes-Pellerin, Nathalie Jolly, Emmanuel Roux, Olivier Schwartz, Sylvie van der Werf, Michael White, Arnaud Fontanet

**Affiliations:** 1 Emerging Diseases Epidemiology Unit, Department of Global Health, Institut Pasteur, Université Paris Cité, Paris, France; 2 Department of Epidemiology, Biostatistics and Occupational Health, Centre for Clinical Epidemiology, McGill University, Lady Davis Institute for Medical Research, Jewish General Hospital, Montreal, QC Canada; 3 Infectious Diseases Epidemiology and Analysis G5 Unit, Department of Global Health, Institut Pasteur, Université Paris-Cité, Inserm U1347, Paris, France; 4 Molecular Genetics of RNA Viruses Unit, Department of Virology, Institut Pasteur, Université Paris Cité, CNRS UMR 3569, Paris, France; 5 Interactomics, RNA and Immunity Unit, Institut Pasteur, Université Paris Cité, Paris, France; 6 Department of Virology, Amiens University Medical Center, Amiens, France; 7 Virus and Immunity Unit, Institut Pasteur, Université Paris Cité, Paris, France; 8 Hôpital de Crépy-en-Valois, Crépy-en-Valois, France; 9 Pôle de Coordination de la Recherche Clinique (PC-RC), Institut Pasteur, Université Paris-Cité, Paris, France; 10 Biobanque de Collections de Ressources Biologiques d’Origine Humaine (ICAReB-Biobank), Center for Translational Science, Institut Pasteur, Université Paris Cité, Paris, France; 11 PACRI, Risques Infectieux et Emergents, Conservatoire National des Arts et Métiers, Paris, France; 12 Agents infectieux résistance et chimiothérapie Research Unit, UR4294, Jules Verne University of Picardie, Amiens, France

**Keywords:** Epidemiology, Risk factors

## Abstract

In this cohort study, we aimed to study the risk of SARS-CoV-2 infection and its association with household exposure, as well as demographic and health factors. Between March 2020 and April 2022, we conducted a cohort study among adults and children aged 5 years or more in a town in northern France. Participants were screened repeatedly for post-infection anti-SARS-CoV-2 immunity. Infection dates were inferred in hierarchical order from symptomatic episodes or virological tests, or if unavailable from the timing of other cases in the household or the window of seroconversion. Household exposure to a case of SARS-CoV-2 during the infectious phase was defined as a time-varying exposure. We included 830 participants with a mean follow-up of 453 days (12,353 total person-months), during which we identified 491 infections (incidence rate 39.7 per 1,000 person-months). In adjusted analyses, exposure in the household to an infected individual was associated with an incidence rate ratio (IRR) of 16.48 (95% CI 12.29–22.09), and baseline statin use was associated with a decreased risk of infection (IRR 0.35, 95% CI 0.13–0.89). These results could contribute to the development of additional prophylactic strategies, e.g., post-exposure, for the population at high risk of severe forms of COVID-19.

## Introduction

Among all possible settings of interaction between a person infected with SARS-CoV-2 and a potential secondary case, households are among the most prone to result in transmission. Estimates of secondary attack rates are higher in households than in other settings^1^. Initial findings reported a secondary attack rate around 17%.^2^ This rate was higher for the more transmissible strains that emerged thereafter, e.g., 29.7% for the Delta variant or 42.7% for the Omicron variant^3^. In a large series of close to 600,000 cases in France from October 2020 to August 2022, 45% of transmission events in which the source case could be identified were reported within the household, making it the predominant setting of transmission^4^. Mitigation of transmission in households may be more challenging than in other settings considering close and prolonged contacts. Households therefore represent a setting of choice to asses transmission dynamics, including how individual characteristics such as demographic characteristics, previous immunity, comorbidities, or chronic medication can affect the risk of transmission^5–8^.

Early during the COVID-19 pandemic, several existing repurposed drugs were tested, mainly in patients infected with SARS-CoV-2 to prevent progression to severe COVID-19. Statins, a hypercholesterolemia treatment indicated in primary or secondary prevention of cardiovascular disease, were soon identified as a candidate given their anti-inflammatory properties and their potential impact on cell entry of SARS-CoV-2 through the limitation of membrane cholesterol content or lipid rafts^9,10^. Several observational studies quickly pointed to a reduced risk of severe COVID-19 outcome associated with prior statin treatment^11,12^. Randomized trials on the other hand did not show any benefit of statins in hospitalized COVID-19 patients^13,14^. The role of statins to reduce the risk of SARS-CoV-2 infection has also been explored in several observational studies that have reported a decreased risk associated with baseline statin use^15–17^. While the burden of SARS-CoV-2 has substantially decreased, many fragile patients remain at risk of severe disease despite vaccination and could benefit from additional forms of prophylaxis. Investigating a potential benefit of baseline statin use on the risk of infection could help design new prophylactic strategies such as post-exposure prophylaxis, considering that SARS-CoV-2 infection has an incubation period of several days, offering a potential window of opportunity for prophylaxis initiation after exposure identification^18^.

In this cohort study, our objective was to estimate the incidence of SARS-CoV-2 infection and its association with demographic characteristics, household exposure, underlying conditions, and baseline statin use.

## Methods

### Study design

Between March 2020 and April 2022, we conducted a cohort study in Crépy-en-Valois, a town of approximately 15,000 inhabitants located around 60 km north of Paris. This town underwent one of the first documented COVID-19 outbreaks in France in early 2020. Between March and May 2020, we conducted several school-centred studies^19^ including children aged 5 years and above and their household members. In November 2020, we expanded the study to include a household-based cohort of local inhabitants beyond the schools where the study initially took place. We invited all participants of these initial studies to participate in the cohort, as well as all people living in the town or nearby, and their household members. All participants or their parents or legal guardians provided informed consent. Informed assent of minors was secured in addition to parents’ consent. A dedicated information document and an illustrated information sheet explaining the study were provided to minors above and below 15 years old, respectively. We included adults as well as children aged 5 years and above, and no exclusion criteria were applied. In another subset of the study, we enrolled staff members (including healthcare workers) of nursing homes and long-term care units of the Crépy-en-Valois hospital, whose sera had been screened for SARS-CoV-2 antibodies by the nearby Centre Hospitalo-Universitaire (CHU) of Amiens in May 2020. For participants in both study groups (households and staff members), visits took place every six months. At each visit, we collected information on socio-demographic characteristics, comorbidities, and medication. We asked about past SARS-CoV-2 infection, including the date of symptom onset and of the positive diagnostic test, as well as the presence of cases within the household, their date of symptom onset and of the positive diagnostic test. These cases could be participants of the study or not, as we did not require participation of all household members for inclusion. We also collected blood samples at each visit to test for the presence of antibodies targeting SARS-CoV-2. The study was registered with ClinicalTrials.gov (NCT04644159) and received ethical approval by the Comité de Protection des Personnes Nord Ouest IV. The initial school-based studies in spring 2020 were registered with ClinicalTrials.gov (NCT04325646) and received ethical approval from the Comité de Protection des Personnes Ile de France III. For all studies, participants did not receive any compensation. All methods were performed in accordance with the relevant guidelines and regulations.

### Serological assays

Sera collected at each visit were tested with three different serological assays. Two were developed by teams of the Institut Pasteur to measure antibodies to the Spike and the Nucleocapsid proteins of SARS-CoV-2. The first assay is a multiplex microsphere-based immunoassay based on the Luminex technology, measuring antibodies against up to 30 antigens including 13 antigens to SARS-CoV-2^20,21^. The second assay is a Luciferase-Linked ImmunoSorbent Assay (LuLISA)^22^. The third assay was conducted by the CHU Amiens with a commercial test to measure anti-Spike antibodies (Wantai SARS-CoV-2 Ab ELISA, Evolis, BioRad^23^; or Chemiluminescence SARS-CoV-2 IgG II Quant assay Alinity, Abbott^24^, except for sera collected in the spring of 2020 for the school-based studies which we tested for anti-Spike antibodies with a flow cytometry-based assay (S-flow)^25^.

Overall, we classified sera as positive for anti-Spike antibodies if they tested positive for at least two of the three methods that were used (Luminex, LuLISA, and S-flow or commercial test), and positive for anti-Nucleocapsid antibodies if they tested positive for at least one of the two methods that were measuring these antibodies (Luminex and LuLISA).

### SARS-CoV-2 infection dates

At each visit, we identified recent SARS-CoV-2 infections through a reported positive diagnostic test (RT-PCR or rapid antigenic test), a reported medically confirmed diagnosis, or anti-SARS-CoV-2 antibody seropositivity. Post-infection seropositivity was defined as the detection of anti-Spike protein antibodies in unvaccinated individuals, and anti-Nucleocapsid protein antibodies in vaccinated individuals. The date of infection was set as the reported date of positive diagnostic test, of the medically confirmed diagnosis, or of associated onset of symptoms if any. For post-infection seropositive participants without positive diagnostic test or medically confirmed diagnosis, we defined the date of infection as the reported date of symptom onset for any symptomatic episode that included at least one of five selected symptoms (fever, cough, shortness of breath, ageusia, anosmia) and occurred in the seroconversion window of the participant. When none of these dates were available, we applied the same infection date as that of another household member for whom the infection date occurred in the same timeframe as the seroconversion window of the participant. When no infection was documented for other household members, the date of infection was set as the midpoint between the last negative and the first positive serological assessment, or between January 1st, 2020 and the first serological assessment when no prior negative assessment was available.

### SARS-CoV-2 secondary cases

We assumed infected participants were infectious for 8 days, starting one day prior to symptom onset, or 3 days prior to the positive diagnostic test for asymptomatic cases^26^. Other household members were considered exposed during this infectious phase, with a lag of 2 days after the beginning and 8 days after its end, to account for the plausible range of incubation periods^18^. For households with infections at uncertain dates (i.e., determined based on the infection date of another household member, or based on the midpoint of the seroconversion window, as described previously), we analysed separately the time intervals during which these infections likely occurred and did not consider them as household exposure. According to our definition of household exposure, people living in single-person households were never considered exposed.

### SARS-CoV-2 variants

We identified the predominant strains in France throughout the course of the study with the sequencing surveys performed weekly on a random subset of positive samples by *Santé Publique France* from early 2021: historical strain prior to January 1st, 2021, Alpha and Beta/Gamma until June 16th, 2021, Delta until December 1st, 2021, Omicron until the end of the study period. The Alpha, Beta, and Gamma variants circulated concomitantly in the first half of 2021. Alpha was notably predominant during that period (Beta and Gamma never represented more than 20% of the circulating strains)^27^.

### Statistical analysis

Participants were censored at the time of infection or at the last visit, whichever occurred first. We considered the follow-up of all participants started on January 1st, 2020. We analysed the factors associated with the risk of SARS-CoV-2 infection through a negative binomial model using generalized estimating equations to account for the longitudinal nature of the data, and robust variance estimators. Each factor of interest was analysed both in univariable analysis and in a multivariable model including all factors potentially associated with the risk of infection based on prior knowledge^28^. These include sex as a binary variable, age at inclusion (in 10-year categories from under 10 to 61 years and above), socio-professional category (categorized as lower, higher, or no professional activity). We included the following underlying conditions as binary variables: cardiovascular disease, chronic respiratory disease, diabetes mellitus, cancer, immunosuppression, and baseline statin use. Given the reliance of self-declared medical information, we decided to classify underlying conditions and baseline statin use as non-time-dependent variables to account for variability in notification quality, that is participants were considered exposed if they reported the condition or baseline statin use at least at one of the visits. We included the COVID-19 vaccine status (defined as the number of doses), the predominant SARS-CoV-2 strain at the time, and the smoking status (non-smokers, active smokers, or former smokers) as time-dependent variables. We performed multiple imputation for variables with missing data (smoking status and socio-professional category) using chained equations based on the other covariates in the model. For the non-time-dependent socio-professional category variable, we imputed the variable at the first point of follow-up for each participant, and assigned the imputed value to all the subsequent time points of the same iteration. We pooled results of five iterations using Rubin’s method.

## Results

Between November 2020 and November 2021, we included 830 participants, including 530 who had participated in the school-centred studies in the spring of 2020. Of those, 107 (12.9%) were included among the nursing home and long-term care unit staff members and the other 723 (87.1%) from the community-based enrolment. The mean follow-up time was 453 days and ranged between 18 and 849 days, amounting to a total of 12,353 person-months. 200 participants (24.1%) were lost to follow-up before the last visit in April 2022 and before any identified infection.

The 830 participants represented 444 households, with a mean number of participants per household of 1.9 (standard deviation 1.2). Most participants reported living in 3-to-4 person households (*n* = 382, 46.1%), while only 33 participants (4.0%) lived in single-person households. The study population was predominantly female (61.7%) and relatively young, reflecting the recruitment process that followed the initial school-based study: median age 43 years (interquartile range 19–52), 176 (21.2%) were aged under 18 years. Active smokers represented 19.2% of the study population. The most frequent comorbidity was cardiovascular disease (12.2%). Twenty-four participants (2.9%) reported treatment with statin. The characteristics at inclusion of the study participants are detailed in Table 1.

**Table 1:** Socio-demographic and medical characteristics of participants at inclusion.

	Total 830 participants *N* (%)
Age at inclusion (years), median (IQR)	43 (19–52)
Female sex	512 (61.7)
Socio-professional category	
Upper	350 (42.2)
Lower	174 (21.0)
Inactive	297 (35.8)
Missing	9 (1.1)
Number of household members	
1	33 (4.0)
2	117 (14.1)
3	133 (16.0)
4	249 (30.0)
5	135 (16.3)
6 or more	42 (5.1)
Missing	121 (14.6)
Smoking status	
Active smoker	87 (10.5)
Former smoker	159 (19.2)
Non-smoker	582 (70.1)
Missing	2 (0.2)
Statin	24 (2.9)
Medical conditions	
Diabetes	28 (3.3)
Chronic respiratory disease	63 (7.6)
Cardiovascular disease	101 (12.2)
Immunodepression	7 (0.8)
Chronic kidney failure	3 (0.4)
History of cancer	21 (2.5)
Dyslipidemia	35 (4.2)
SARS-CoV-2 infection	491 (59.2)
Predominant strain at the time of infection	N (% of infections)
Historical strain	284 (57.8)
Alpha and Beta/Gamma	52 (10.6)
Delta	13 (2.7)
Omicron	142 (28.9)
Identification of the date of infection	N (% of infections)
Date of RT-PCR, antigen test, or medical diagnosis	243 (49.5)
Date of symptom onset	166 (33.8)
Date of SARS-CoV-2 circulation in the household	52 (10.6)
Midpoint between visits or between January 1st, 2020 and first visit	30 (6.1)

We observed 491 infections (incidence rate 39.7 per 1,000 person-months), among which 243 (49.5%) were confirmed based on a reported positive RT-PCR, rapid antigen test, or practitioner diagnosis, and 248 (50.5%) were identified based on seropositivity only (anti-Spike antibodies in unvaccinated participants, anti-Nucleocapsid antibodies in vaccinated participants). Among the latter, the date of infection was determined by the date of symptom onset for 166 participants (33.8%) and inferred based on the date of SARS-CoV-2 circulation in the household for 52 (10.6%) or as the midpoint between two serological assessments for 30 (6.1%).

We observed household exposure in 252 participants, representing 119.3 person-months of exposure, during which 75 infections (15.3% of all infections) were observed. The incidence rate ratio (IRR) of household exposure was 22.46 (95% confidence interval 17.17–29.38). We observed a higher risk of infection in women (IRR 1.23, 95% CI 1.02–1.47) and a lower risk in current smokers (IRR 0.65, 95% CI 0.48–0.90). The risk did not differ significantly between age groups. We observed 9 infections among the 24 participants treated with statin at baseline, with an IRR for statin exposure of 0.41 (95% CI 0.19–0.85). In a multivariable model including age, sex, socio-professional categories, medical conditions, smoking status, vaccine status, and the predominantly circulating strain, household exposure (IRR 16.48, 95% CI 12.29–22.09) and female sex (IRR 1.53, 95% CI 1.23–1.91) remained associated with an increased risk of infection, while the risk associated with underlying cardiovascular disease increased to an IRR of 1.58 (95% 1.12–2.26); current smoking status (IRR 0.66, 95% CI 0.45–0.97) and baseline statin use (IRR 0.35, 95% CI 0.13–0.89) remained associated with a decreased risk of infection (Table 2).

**Table 2:** Incidence rate ratios of sociodemographic characteristics, household exposure, smoking status, vaccine status, medical conditions, and treatment with the risk of SARS-CoV-2 infection.

	Number of participants with SARS-CoV-2 infections	Person-months exposed	Incidence rate (per 1,000 person-months)	Incidence rate ratio (univariable analysis)	Incidence rate ratio (multivariable analysis)
Age (years)					
5–10	33	972.8	33.9	1 (ref)	1 (ref)
11–20	93	2196.3	42.3	1.15 (0.83–1.61)	1.23 (0.81–1.85)
21–30	22	541.5	40.6	1.18 (0.76–1.83)	1.52 (0.77–2.97)
31–40	78	1682.0	46.4	1.36 (0.97–1.90)	1.81 (0.95–3.46)
41–50	133	3452.5	38.5	1.06 (0.78–1.45)	1.12 (0.60–2.07)
51–60	91	2384.0	38.2	0.95 (0.67–1.35)	1.31 (0.69–2.47)
61 and above	41	1123.5	36.5	0.90 (0.59–1.38)	1.36 (0.77–2.41)
Sex					
Male	173	4923.2	35.1	1 (ref)	1 (ref)
Female	318	7429.4	42.8	**1.23 (1.02–1.47)**	**1.53 (1.23–1.91)**
Socio-professional category					
Inactive	171	4334.7	39.4	1 (ref)	1 (ref)
Higher	210	5307.8	39.6	1.01 (0.83–1.22)	0.99 (0.60–1.62)
Lower	105	2538.8	41.4	1.01 (0.79–1.29)	1.21 (0.72–2.04)
Missing^a^	5	171.3	29.2	-	-
Household SARS-CoV-2 exposure^b^					
No	313	11910.8	26.3	1 (ref)	1 (ref)
Yes	75	119.3	628.7	**22.46 (17.17–29.38)**	**16.48 (12.29–22.09)**
Smoking status					
Non-smoker	351	8254.6	42.5	1 (ref)	1 (ref)
Active smoker	42	1443.1	29.1	**0.65 (0.48–0.90)**	**0.66 (0.45–0.97)**
Former smoker	94	2336.3	40.2	0.91 (0.72–1.14)	0.97 (0.74–1.27)
Missing^a^	4	48.7	82.1	-	-
Main circulating strain					
Historical strain	284	6789.0	41.8	1 (ref)	1 (ref)
Alpha and Beta/Gamma	52	2141.4	24.3	**0.50 (0.38–0.66)**	**0.66 (0.48–0.90)**
Delta	13	2200.5	5.9	**0.11 (0.07–0.20)**	**0.21 (0.11–0.41)**
Omicron	142	1221.7	116.2	**2.48 (2.04–3.01)**	**3.72 (2.28–6.06)**
COVID-19 vaccine status					
Unvaccinated	363	9699.4	37.4	1 (ref)	1 (ref)
1 dose	10	399.9	25.0	0.58 (0.32–1.05)	1.52 (0.72–3.22)
2 doses	51	1648.8	30.9	**0.73 (0.55–0.96)**	**0.51 (0.29–0.89)**
3 doses	67	604.5	110.8	**2.75 (2.12–3.55)**	0.62 (0.36–1.07)
Baseline statin use	9	434.7	20.7	**0.41 (0.19–0.85)**	**0.35 (0.13–0.89)**
Underlying conditions					
Cardiovascular disease	64	1415.5	45.2	1.12 (0.84–1.48)	**1.58 (1.11–2.26)**
Diabetes	20	422.0	47.4	1.21 (0.78–1.88)	1.48 (0.85–2.56)
Chronic respiratory disease	39	978.3	39.9	0.93 (0.66–1.30)	1.06 (0.72–1.54)
Immunosuppression	6	87.6	68.5	1.78 (0.70–4.56)	1.41 (0.61–3.26)
History of cancer	13	315.2	41.2	1.11 (0.62-2.00)	0.95 (0.54–1.68)

Incidence rate ratios based on univariable and multivariable negative binomial regression models accounting for longitudinal analysis through generalized estimating equations. Incidence rate ratios with 95% confidence intervals excluding 1 are in bold. The multivariable model contains all variable shown in the table. Statins and variables pertaining to medical conditions are binary. A: multiple imputations for the incidence rate ratio; b: time periods likely including infections but with uncertain infection dates were analysed separately (data not shown) and were not considered as household exposure.

## Discussion

In this community-based cohort study conducted over two years among adults and children in northern France, we identified that 59% of participants were infected at least once through follow-up. Close to 15% of these infections occurred during a period when at least another household member was infected, which was associated with an incidence rate ratio of 16.48 (95% CI 12.29–22.09). The risk was consistent among age groups but was higher in women, people with underlying cardiovascular disease, and lower in current smokers. We also found that participants with baseline statin use had a decreased risk of infection with an IRR of 0.35 (95% CI 0.13–0.89), after accounting for sociodemographic and comorbidity confounders as well as household exposure.

The strong increase in risk associated with household exposure is consistent with past studies that report that household is the setting where contacts are most likely to result in transmission and the most frequently reported setting of transmission in case series^1,4,29^. In the present study, we provide a different approach to measuring the risk of household transmission, indicating that people are approximately 16 times more likely to be infected when one of their household members is infected, than at other time points. The interpretation of this estimate is relatively straightforward and could help public health authorities in their messaging to decision makers and the public regarding the benefit of in-home isolation. Such mitigation strategies are relevant for SARS-CoV-2 but are also crucial for future emerging respiratory viruses which might share transmission properties with SARS-CoV-2.

The reduced risk of infection associated with active smoking has been reported in other settings^30,31^. The association might be confounded by better compliance with ventilation of indoor areas among smokers, which is associated with a lower risk of household SARS-CoV-2 transmission^32^. It might also reflect different contact patterns among smokers who may avoid indoors interactions to limit exposure to smoke for others. Regardless of the true underlying causality, smoking has been consistently associated with poor outcomes in patients with COVID-19^33,34^.

The increased risk of infection in women has not been documented in seroprevalence surveys in France^35^. In a meta-analysis on household transmission, the risk of transmission was not associated with the sex of the index case or of the contact person^3^. Our study enrolment being based on school communities, the population skews towards multigenerational households with children. In this setting, a hypothetical explanation is that contact patterns with children differ between men and women in French households^36^. The consistent risk among age groups further confirms the similar susceptibility of children to SARS-CoV-2 infection compared with adults, as has been shown across several seroprevalence studies^37,38^.

It is unclear if the decreased risk of infection in baseline statin users reflects a true biological effect or if it results from confounding by healthcare-related behaviours. The efficacy of statins in patients hospitalized for COVID-19 remains uncertain^13,14^, although a recent meta-analysis on 7 randomized trials has reported a 12% reduction of the case-fatality rate^39^. Regarding the effect of statins on the risk of infection, several studies have produced discrepant results. A large study in Sweden using medico-administrative datasets compared 127,542 statin users with 445,153 non-users and found a hazard ratio of 0.90 (95% CI 0.85–0.94) in statin users for the risk of COVID-19 diagnosis^16^. Similarly, a case-control study in Spain found an odds ratio of RT-PCR-confirmed COVID-19 of 0.94 (95% CI 0.90–0.99) for statin exposure^15^. On the other hand, a secondary analysis of a randomized trial of pitavastatin in people living with HIV found no change in the risk of infection compared to placebo^40^. Our IRR of 0.39 is lower than the cited estimates, but the wide 95% confidence interval (upper limit at 0.89), which reflects our small sample size (24 participants exposed), suggests that the effect, if causal, would rather be in the upper range of this confidence interval. One of the main strengths of our study was the use of highly sensitive and specific serological assays, allowing better case detection than if based on the often symptom-driven RT-PCR tests. Epidemiology is however familiar with non-causal associations of statins with numerous outcomes^41^. Hence our findings warrant confirmation in different settings, using different designs. As the options for post-exposure prophylaxis remain limited, we believe our findings can contribute to the design of such a pharmaceutical intervention. Households seem an appropriate setting to evaluate post-exposure prophylaxis, as has been done before, considering the high risk of transmission documented in this and other studies, and the easy identification of contact persons^42^.

Our infection detection strategy based on a combination of retrospective collection of positive tests, medical diagnoses, and serological assessments every six months significantly reduces the risk of missing infection cases. Our longitudinal analysis allowed us to finely adjust for household exposure. The limitations of the study include limited information on the infection prevention and control measures implemented in households. We cannot rule out unmeasured confounding, e.g., people taking statins, a preventive treatment, might have been more compliant with prevention measures if their perceived risk of severe COVID-19 was higher given cardiovascular disease. The fact that the association remained largely unchanged after adjustment on underlying conditions (including cardiovascular disease) suggests little confounding based on differences in underlying fragility. While we designed a prospective cohort study, we had to retrospectively reconstruct transmission chains in the household, with some uncertainties regarding some infection dates, and sometimes analysing information on household members who did not participate in the study. A potential misclassification of some household exposures could result from infections occurring while household members being away from home, a subtlety we did not measure. Other potential sources of unmeasured confounding include exposures in the community such as attending gatherings or visiting high-density settings. However, these were probably rare during household exposure as in-home isolation was recommended for household contacts until early 2022 in France. The high proximity in households compared to most community settings also supports the hypothesis that most transmission events occurred in the household. Health-related information was self-declared and thus potentially inconsistently identified from visit to visit. To increase our ability to detect underlying conditions and medications, we chose to include these variables as non-time-varying, potentially resulting in some misclassification, albeit likely limited. A related limitation is that most participants were unable to indicate the exact statins they were taking, hence we had to group all statins together in our analyses. Other studies have reported potentially differential effects between statins^15^, which warrant further investigation. The conduction of the study in a small town in France limits the generalizability to other settings, e.g., other countries or dense urban environments. Finally, 24% of participants were lost to follow-up before infection. It is unclear how this could have biased our findings on exposures of interest such as household exposure, sex, or baseline statin use.

This cohort study provides further insight on the major role of households in SARS-CoV-2 transmission across all age groups, particularly for women. The decreased risk associated with baseline statin use, given methodological limitations, warrants confirmation in different settings, but could help design future prophylaxis strategies, e.g., post-exposure prophylaxis. Households are an appropriate setting to evaluate such strategies.

## Data Availability

All data produced in the present study are available upon reasonable request to the authors (corresponding author at simon.galmiche@mail.mcgill.ca).
